# Drug-related relapses in drug reaction with eosinophilia and systemic symptoms (DRESS)

**DOI:** 10.1186/s13601-020-00359-2

**Published:** 2020-11-23

**Authors:** Lukas Jörg, Arthur Helbling, Daniel Yerly, Werner J. Pichler

**Affiliations:** 1 Department of Rheumatology, Immunology and Allergology, Inselspital, Bern University Hospital, University of Bern, 3010 Bern, Switzerland; 2 ADR-AC GmbH, Adverse Drug Reactions, Analysis and Consulting, Bern, Switzerland

**Keywords:** Drug hypersensitivity, Drug allergy, Multiple drug hypersensitivity syndrome (MDH), Flare-up reaction, Relapse, Drug reaction with eosinophilia and systemic symptoms (DRESS), T-cell, Eosinophilia, Drug induced hypersensitivity syndrome (DiHS)

## Abstract

**Background:**

A drug reaction with eosinophilia and systemic symptoms (DRESS) is a severe T cell mediated hypersensitivity reaction. Relapses of symptoms in the recovery phase are frequent and linked to the reduction of the corticosteroid treatment, to viral reactivations or to the exposure to new drugs. Here, we analyzed, how often the exposure to new drugs leads to new sensitization or drug-related relapses without detectable sensitization.

**Methods:**

46 patients with DRESS treated in the allergy division of the Inselspital, Bern University Hospital, were retrospectively assessed. Drug-related relapses were analyzed in terms of frequency and whether a possible sensitization evaluated by skin tests and/or lymphocyte transformation tests (LTT) to the new drugs was detectable. Furthermore, drug tolerance was evaluated in a subset of patients.

**Results:**

56 relapses were observed in 27 of 46 patients with DRESS (58.7%). 33 (58.9%) of these relapses were associated with the use of new drugs, 30 drug-related relapses were evaluated by patch test and/or lymphocyte transformation test. In 8/30 (26.7%) drug-related relapses, a sensitization to the new drug was demonstrated, suggesting the emergence of a multiple drug hypersensitivity syndrome (MDH). 14 patients experienced 22 drug-related relapses without any detectable sensitization and only 1/6 patients developed new symptoms upon reexposure.

**Conclusion:**

Patients with DRESS frequently suffered from drug related relapses. Half of the patients with drug-related relapses developed a MDH with proven sensitizations not only to the DRESS inducing drugs, but also to newly applied drugs. When not sensitized, drugs involved in drug related relapses could be reintroduced, if needed. Here, we propose a procedure for drug testing and future management of drug-related relapses in DRESS.

## Introduction

Drug reactions with eosinophilia and systemic symptoms (DRESS), also called drug induced hypersensitivity syndrome (DiHS) are severe T cell mediated drug hypersensitivity reactions (DHRs), leading to exanthema, fever, eosinophilia, lymphadenopathy, and hepatitis [1–3]. Other organs such as the kidneys, heart, lungs, pancreas, bone marrow, or the cerebral areas are affected occasionally [2, 3]. The clinical recognition of DRESS is challenging. Especially in the prodromal stage, non-specific symptoms resembling those of infections or autoimmune diseases may appear [4]. Furthermore, the latency period between exposure and symptoms is usually long (2–8 weeks) [5]; consequently drugs as potential triggers are often not considered. However, shorter latency periods (< 14 days) have been observed [6]. Despite discontinuation of the causing drug, clinical improvement is often delayed [7]. DRESS may persist for weeks or sometimes even for months [8]. Relapses manifesting as exacerbations of exanthema, recurrent eosinophilia, or hepatitis are typical, even if the inducing drug is discontinued [4, 9]. Relapses may be related either to viral reactivations [7, 10], or to rapid reduction of systemic steroids [4, 11], or to administration of new drugs or to previously tolerated drugs after dose increase [4, 12–14].

Picard et al. find in their study that 25% of the DRESS patients had drug-related relapses [13]. However, they did not evaluate if these patients were sensitized to the causing drug. Drug-related relapses, for which sensitization to the newly introduced drug has not been proven may be associated with an unknown mechanism such as stimulation of already activated T cells. In these cases patients may tolerate the suspected drug after complete recovery from the relapse. On the other hand, some patients showed a new T cell sensitization to the causing drug, leading to a multiple drug hypersensitivity syndrome (MDH) [14–16]. These patients are at risk of developing a drug hypersensitivity on re-exposure [17], therefore, they have to avoid multiple structurally different drugs. Thus, the distinction between drug-related relapse without proven sensitization and MDH is pivotal for the subsequent therapeutic management.

The aim of this study was to assess the frequency of drug-related relapses and to evaluate whether skin tests and/or lymphocyte transformation tests are useful in drug-related relapses to identify patients with MDH.

## Methods

### Study design

This study was a monocentric, retrospective analysis of patients with DRESS, examined between January 2011 and December 2018 at the out-patient allergy division of the Inselspital, Bern University Hospital, Switzerland. All data were obtained in September 2019 using a search tool from the hospital record database. DHR cases listed as “DRESS”, “drug reaction with eosinophilia and systemic symptoms”, “drug rash with eosinophilia and systemic symptoms”, “drug induced hypersensitivity syndrome”, “DiHS” and the combined terms “hypersensitivity”, "exanthema", "eosinophilia" and "fever" were evaluated. All patients included in this study gave informed consent for study participation and data publication. The study was approved by the local ethics committee (Kantonale Ethikkommission Bern).

### Study population

All DRESS cases were evaluated based on the Regiscar scoring system [18], which classifies a DRESS as definitive case (≥ 6 points), probable case (4–5 points), possible case (2–3 points) or no case (< 2 points). Only patients with a probable or definite DRESS with ≥ 4 points and a complete allergy workup (skin tests and/or lymphocyte transformation test (LTT) to involved drugs) were included. Skin tests and LTT were performed during the recovery state within 24 months after diagnosis DRESS. Skin test results were evaluated according to the EAACI/ENDA guidelines [19]. Since intradermal tests carry the risk of triggering a relapse in DRESS, all skin tests were performed using patch tests [20]. Although patch tests may have a low risk of relapse, they are generally considered to be safe in severe DHR [20]. For drugs with limited information on non-irritant test concentrations, 10–30% dilutions in petrolatum were used for patch test according to the guidelines of the European Society of Contact Dermatitis [21] (Additional file 1: Table S1). Exclusion criteria for this study were pre-existing eosinophilic skin disorder, confirmation of another diagnosis, which could explain the DRESS like symptoms, incomplete cases, a refusal to participate, and missing follow up at our allergy division.

### Patient assessment

For each DRESS case, the following information have been recorded and analyzed: clinical features (e.g. exanthema, fever, eosinophilia, presence of atypical lymphocytes, organ involvement), administered drugs, latency period, comorbidities, the occurrence of relapses in the course and after the DRESS period, and results of skin test and lymphocyte transformation test (LTT). All cases were assigned to either one of two groups:

- Group 1: DRESS, without proven drug-sensitization(s).
- Group 2: DRESS with proven drug-sensitization(s) to inducing drug.

**Fig. 1:**
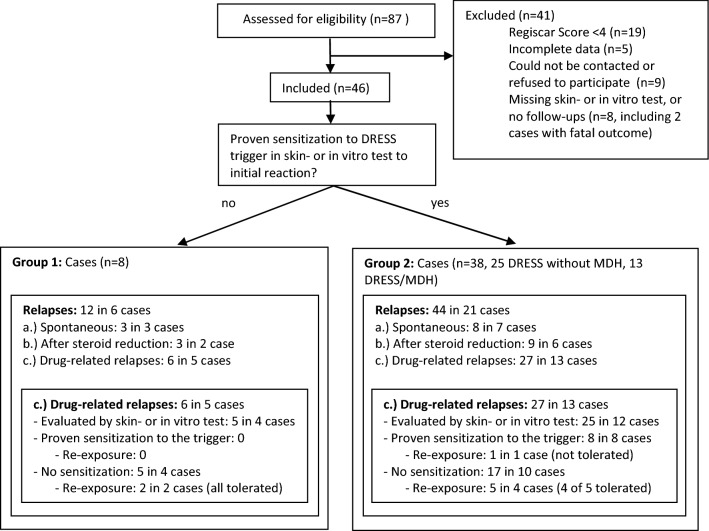
Study procedure and patient allocation. DRESS cases were assigned to one of two groups: group 1: DRESS without proven drug sensitization and group 2: DRESS with proven drug sensitization to inducing drug. Frequency of drug-related relapses (subgroups a-c), skin tests and/or LTT to possibly involved drugs were evaluated in each group. Patients who were re-exposed to the triggering drug after the drug-related relapse resolution were identified

In each group the focus was on relapses occurring during or within 24 months after resolution of DRESS. The frequency of drug-related relapses and skin tests and/or LTT to possibly involved drugs was investigated. Patients who were re-exposed to the triggering drug after the relapse resolution were identified. The detailed procedure is summarized in a flowchart (Fig. 1).

MDH was defined as an immune mediated DHR to two or more unrelated drugs confirmed either by skin or by in-vitro tests [14, 15]. A relapse was defined as a transient re-occurrence of clinical symptoms and/or laboratory signs during or following the initial DHR (such as exanthema, recurrent eosinophilia, elevation of liver enzymes). All relapses were evaluated for a temporal relationship to the administration of new drugs or the reduction of systemic steroids. They were divided into three subgroups based on possible causes: (a) spontaneous forms without obvious cause; (b) relapses within two days after systemic steroid reduction; (c) relapses in connection with the administration of new drugs or dose increase of a previously tolerated drug (within three days). Viral re-activations were evaluated only in a minority of subjects and were not considered in this classification of relapses.

### Statistical analysis

Analyses were performed using Graphpad Prism 8 (GraphPad Software, Inc, La Jolla, CA, USA). All results were summarized using descriptive statistics. Proportions were expressed in percentage; several continuous variables (eg. age, latency period etc.) were reported as mean and standard deviation or median and interquartile ranges.

## Results

### Patient characteristics

87 patients with DRESS were identified in our database (Fig. 1). 19 of these patients achieved a Regiscar score of less than 4 and were thus excluded. Most of these excluded cases had severe forms of maculopapular exanthema (MPE) with fever, eosinophilia, and facial swelling without organ involvement or a duration of DRESS ≤ 14 days. Five subjects were excluded due to incomplete data, nine could not be contacted or refused to participate. Six patients were excluded due to missing or incomplete allergy workup or follow-up, and two patients had a lethal outcome. 46 patients fulfilled all criteria and were evaluated (Table 1). The most frequently involved organ was the liver (39/46, 84.8%), followed by the kidneys (12/46, 26.1%) and the bone marrow (cytopenia; 6/46, 13.0%). The mean peak eosinophilia level was 3.0 G/L (± 3.5). Comorbidities were infectious (15/46, 32.6%), cardiac (9/46, 19.6%), and autoimmune diseases (8/46, 17.4%). The median latency from the first drug intake to the occurrence of symptoms was 19 days (Range 2–90 days). For 11/46 patients (23.9%), the latency was below 10 days.

**Table 1:** Patient characteristics

	Total
	N = 46
Demographics	
Age (at time of DRESS)	52.0 (38.3; 65.0)
Gender (female)	23 (50.0%)
Clinical	
Exanthema (n)	46 (100.0%)
Centro-facial oedema (n)	15 (32.6%)
Fever > 38.0 ºC (n)	35 (76.1%)
Eosinophilia > 0.5G/L (n)	40 (87.0%)
Eosinophilia, mean (G/L)	3.0 ± 3.5
Atypical lymphocytes (n)	20 (43.5%)
Hepatitis (n)	39 (84.8%)
Lymphadenopathy (n)	12 (26.1%)
Renal involvement (n)	12 (26.1%)
Pulmonary involvement (n)	2 (4.3%)
Cardiac involvement (n)	2 (4.3%)
Cytopenia (n)	6 (13.0%)
Other organ involvement^a^	7 (15.2%)
Number of organs involved (mean)	1.4 ± 0.7
Viral reactivation, detected in 8/13 (n)	8 (17.4%)
Lethal outcome (n)^b^	0 (0.0%)
DRESS with MDH	13 (28.1%)
Latency period (days)	19.0 (9.3; 27.8)
Comorbidities	
Autoimmune disease (n)	8 (17.4%)
Renal insufficiency (n)	6 (13.0%)
Infectious disease (n)^c^	15 (32.6%)
Cardiac disease (n)	9 (19.6%)
Epilepsy (n)	8 (17.4%)
Neoplastic disease (n)	7 (15.2%)

Values are median and interquartile ranges (IQR) for continuous variables. Categorical variables reported as n (%)

Multiple drug hypersensitivity syndrome (MDH), drug reaction with eosinophilia and systemic symptoms (DRESS)

^a^Pancreatitis, gastrointestinal involvement, neurological involvement, myositis

^b^2 cases with lethal DRESS outcome were excluded because of missing skin test and lymphocyte transformation test (see Fig. 1)

^c^Refers to the period of one month before the onset of DRESS: 5 × pneumonia, 2 × acute prostatitis, 2 × endocarditis, 1 × septic arthritis, 1 × osteomyelitis, 1 × acute cholecystitis, 1 × cellulitis, 1 × acute toxoplasmosis, 1 × acute rhinosinusitis

Eight out of 46 patients (17.4%) were classified into group 1 without proven sensitization to a DRESS trigger: Seven out of eight patients were evaluated by skin test and LTT (one case without association to drugs). A causative drug inducing DRESS was identified in 38 patients (82.6%) (group 2) including 13 cases with DRESS/MDH (13/46, 28.1%). These 13 patients had been described earlier [14]. Of the 38 cases with proven sensitization, nine and five were evaluated only by skin test and LTT, respectively. In 24 patients both tests were performed, from which 13 had matching results. In six cases only the LTT and in two cases only the skin test revealed a sensitization. Discrepant results were obtained in three cases (Additional file 1: Table S2).

### Relapses in DRESS

**Fig. 2:**
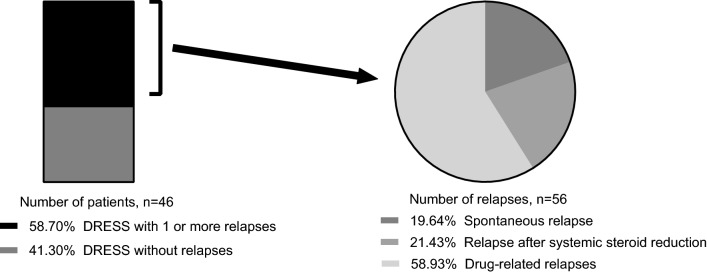
**a** Number of patients with flare-up reactions. **b** Form of flare-up reactions. A total of 56 relapses in 27 of 46 patients have been recorded. Drug reaction with eosinophilia and systemic symptoms (DRESS)

In 27/46 (58.7%) patients with DRESS 56 relapses were observed. 12 occurred after systemic steroid reduction (21.4%) and 11 were spontaneous without an obvious cause (19.6%). Drug-related relapses occurred in 18 patients (18/46, 39.1%) comprising 33 episodes (58.9%) (Fig. 2). Relapses were equally frequent in both groups (group 1: 50.0% of subjects; group 2: 55.3% of subjects). Drug-related relapses were observed more frequently in group 1 (5/8, 62.5% vs. 13/38 34.2%; p = 0.136).

An allergy workup with the drugs involved in the relapses was conducted in 16 patients (4 of subgroup 1c and 12 of subgroup 2c) with 30 drug-related relapses (Fig. 1, Additional file 1: Table S2). All drugs involved in relapses were different to the initial drugs causing DRESS. In eight patients from subgroup c, a sensitization to a suspected and culprit drug was shown (8/16, 50.0%): radio-/gadolinium based contrast agents (4), vancomycin (1), valproic acid (1), betalactam antibiotic (1), and a non-steroidal anti-inflammatory drug (NSAID) (1). No sensitization was found in the remaining drug-related relapses (14 patients with 22/30 drug related relapses, 73.3%).

### Subsequent tolerance of drugs that were involved in drug-related relapses

Of the 14 patients of subgroup c with a drug-related relapse without proven sensitization, six subjects with seven corresponding relapses needed the suspected drug and were re-exposed: six re-exposures (two patients of subgroup 1c and four patients of subgroup 2c) were well tolerated: betalactam antibiotics (2), proton pump inhibitors (2), vancomycin (1) and paracetamol (1). In one patient (subgroup 2c; DRESS trigger: amoxicillin and ceftriaxon), the administration of vancomycin led to a generalized MPE after one day despite negative skin test (Additional file 1: Table S2).

One patient with a flucloxacillin-induced relapse (subgroup 2c; DRESS trigger: carbamazepine) and a documented sensitization by skin test and LTT (sensitized to penicillins and cephalosporins) was accidentally re-exposed to flucloxacillin, cefuroxime and ceftriaxon leading to a generalized MPE following each re-exposure (Additional file 1: Table S2).

### Trigger of DRESS and drug related relapses

**Fig. 3:**
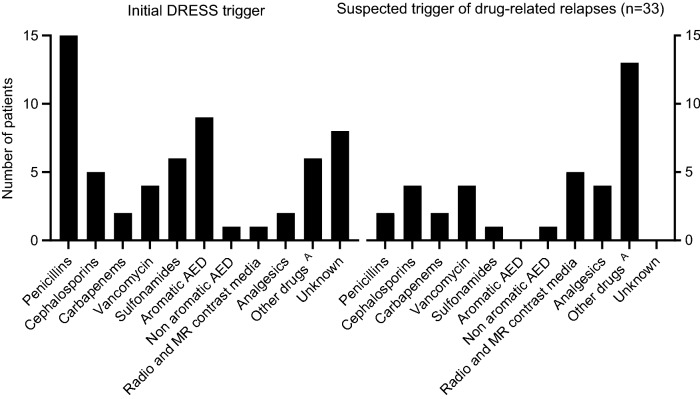
Summary of all triggers of DRESS. All values are reported as n. Drug reaction with eosinophilia and systemic symptoms (DRESS), antiepileptic drug (AED). ^A^DRESS trigger: 5-amino salicylic acid*,* clarithromycin, dabrafenib, darunavir, pantoprazole, rifampicin. Suspected triggers of drug-related relapses: Clindamycin, daptomycin, fluconazole, proton pump inhibitors, quinolones, radio contrast media, zonisamid

The most common drugs inducing DRESS with documented sensitization (not relapses) were penicillin antibiotics (especially piperacillin and amoxicillin; 15/46, 32.6%), followed by aromatic anti-epileptics (9/46, 19.6%) and sulfonamides (6/46, 13.0%). Other triggers were cephalosporins, vancomycin and carbapenems (Fig. 3). Of the 11 patients with latency periods of ≤ 10 days, antibiotics were identified as trigger in nine cases, from which seven were betalactam antibiotics.

Seven of the eight DRESS cases without proven sensitization to a trigger (group 1) occurred after treatment with new drugs: in four cases, antibiotics were suspected to be the trigger: piperacillin/tazobactam (3), minocycline (1).

Drugs involved in drug-related relapses were beta-lactam antibiotics (8/33, 24.2%), vancomycin (4/33, 12.1%), radio- or gadolinium-based-contrast agents (5/33, 15.2%) and analgesics (4/33, 12.1%) (Additional file 1: Table S2).

## Discussion

In more than half of our DRESS cases, a relapse was found. A drug-related relapse was observed in 39.1% of patients. The proportion of cases experiencing drug-related relapses is higher than in the study of Picard et al. [13], probably because we recorded also those relapses, occurring during active DRESS. The majority of the relapses were related to new drugs. Only in a few cases, symptoms emerged spontaneously or after reduction of systemic steroids. How often viral re-activations were involved in relapses cannot be assessed based on our data, since this was only evaluated in 13/46 patients.

There is one important question for the clinician: is it possible to administer drugs previously being involved in relapses again? 8/46 of our patients (17.4%) had drug-related relapses with proven sensitization and were thus classified as MDH. Interestingly, these patients were exclusively from group 2 with a proven sensitization to a DRESS trigger. The total number of MDH cases was 13/46 (28.2%), from which three subjects did not develop drug-related relapses (Additional file 1: Table S2). However, no sensitization to the involved drug was found in the majority of the drug-related relapses. In six out of seven skin test and/or LTT negative drug-related relapses, re-exposure to the triggering drug was well tolerated. Nevertheless, the limited sensitivity of the skin test and LTT needs to be taken into consideration. Unfortunately, the underlying mechanism of skin test or LTT negative drug-related relapses remains unknown. Although a stimulation of already activated T cells may be assumed without persistent sensitization [15], a missing causal relationship cannot be excluded.

Although rarely mentioned in the literature [22–24], betalactam antibiotics, especially penicillin antibiotics such as amoxicillin and piperacillin were the most common triggers in our DRESS cases. Usually, high doses of penicillins are administered (up to 13.5 g/d!), which may promote the development of DHR and relapses [14, 15]. Interestingly, the administration of betalactam antibiotics was associated with a relatively short latency (of < 10 days), an observation that was already described previously [6, 24].

The drugs involved in drug-related relapses are frequently used in DRESS patients. Apart from beta-lactam antibiotics, radio-contrast media, proton pump inhibitors, and analgesics have been relatively frequent involved in relapses.

Whether DRESS cases with and without proven sensitization to the initial trigger are based on the same pathomechanism is still unclear. If sensitization is proven, a drug-specific T cell reaction can be assumed. In case of a missing sensitization in skin test and/or LTT, a false negative test result is possible. However, another (unspecific) pathomechanism has to be considered. It is known that in particular penicillins can promote the re-activation of viruses [12] and are therefore capable of inducing a relapse. We found no significant differences in the frequency of relapses between the two groups. However, there is a trend towards increased drug-related relapses in group 1 (patients without proven sensitization to a DRESS inducing drug). In contrast, a sensitization to drugs involved in relapses was only observed in group 2 (patients with a proven sensitization to a DRESS inducing drug). Our data suggest that drug-related relapses in DRESS cases without proven sensitization are unlikely to lead to a persistent sensitization.

### Is reexposure reasonable in drug related relapses?

When evaluating drug allergy, not only the initial DRESS trigger should be considered, but also drugs that were administered during the active phase of DRESS, if involved in relapses. Santiago et al. have recently shown that a large proportion of DRESS patients develop a new sensitization to antibiotics administered during DRESS [17]. Our data suggest that also other non-antibiotic drugs may cause relapses with detectable sensitization such as radio contrast agents, proton pump inhibitors and NSAID. We therefore propose the following procedure for drug testing and future management:

- 1. Verification of all potential drugs causing DRESS by skin test and/or LTT. In vitro or patch tests (instead of intradermal test with late reading) should be performed to avoid the risk of relapses.
- 2. To discriminate between flare-up and MDH, all suspected drugs in subsequent relapses need to be tested, especially when administered temporarily during the active DRESS phase, e.g. radio contrast agents, NSAIDs, proton pump inhibitors and antibiotics.
- 3. In case of negative tests, reintroduction of these drugs may be allowed when needed, particularly in DRESS without proven sensitization. Proton pump inhibitors appear to be well tolerated. However, this approach bears a small risk of a relapse, which seems acceptable if index symptoms of the relapse were mild.
- 4. Alternatively, a preceding intradermal test and re-exposure by graded challenge under clinical control can be considered. However, these patients who have just experienced a severe DHR, are understandably reluctant to challenge tests just for diagnosis.

The retrospective design and the fact that not all drug-related relapses were tested are obvious limitations of the study. Re-exposures in test negative relapses were performed in only six patients (seven relapses). Furthermore, a selection bias could play a role. Since our data are mainly based on skin tests and LTT, false positive and false negative results are possible. However, especially the LTT is considered an adequate diagnostic tool for drug causality in DRESS [25].

Our observations need to be investigated in a larger, prospective study: all triggers that are involved in drug-related relapses, especially antibiotics, should be systematically tested by skin test and LTT in consideration of a re-exposure in case of a negative result.

## Conclusion

Patients with DRESS are at risk that new drugs may result in another severe DHR. It is therefore imperative to better understand and possibly avoid a MDH course. Our data show that drug-related relapses and MDH were common complications in DRESS. Most drug-related relapses were not linked to a detectable immune response. Involved drugs can be administered again, when not sensitized. To avoid development of a MDH, it is advisable to be as restrictive as possible with administration of new drugs during a DRESS reaction. All drugs involved in relapses should be tested, even when applied later in the DRESS course.

## Supplementary information

**Additional file 1.** Additional Tables.

## Data Availability

The complete datasets used and analysed during the current study are available from the corresponding author upon reasonable request.

## Abbreviations

DHR: Drug hypersensitivity reaction
DiHS: Drug induced hypersensitivity syndrome
DRESS: Drug reaction with eosinophilia and systemic symptoms
MDH: Multiple drug hypersensitivity syndrome
MPE: Maculopapular exanthema
NSAID: Non-steroidal anti-inflammatory drugs
SCAR: Severe cutaneous adverse reactions
