# Supplementary material for: Drug-related relapses in drug reaction with eosinophilia and systemic symptoms (DRESS)
Source: Clin Transl Allergy. 2020 Nov 23;10:52. doi: 10.1186/s13601-020-00359-2 (PMC7682085; doi:10.1186/s13601-020-00359-2)
Supplement: Supplementary file 1 — Additional file 1. Additional Tables. [file 13601_2020_359_MOESM1_ESM.docx]

Table S1 Test conditions of drugs with limited information on non-irritant test concentrations

| **Tested drug** | **Allergen concentration in patch test** |
| --- | --- |
| Clarithromycin | 10% in petrolatum |
| Clindamycin | 10% in petrolatum |
| Dabrafenib capsule | 30% in petrolatum |
| Daptomycin | 10% in petrolatum |
| Darunavir tablet | 30% in petrolatum |
| Fluconazole | 10% in petrolatum |
| Vancomycin | 10% in petrolatum |

Table S2

| Patient | Initial trigger of DRESS | Skin test | LTT | Spont. relapses | Relapses after steroid reduction | Drug related relapses | Suspected trigger of drug-related relapse | Skin test | LTT | Reexposition |
| --- | --- | --- | --- | --- | --- | --- | --- | --- | --- | --- |
| 1 | No proven sensitization Piperacillin/  tazobactam suspected | - | - | 0 | 0 | 1 | Paracetamol | - | n/a | Paracetamol reexposed (tolerated) |
| 2 | No proven sensitization Piperacillin/  tazobactam suspected | - | - | 0 | 0 | 1 | Cefepime | - | - |  |
| 3 | No proven sensitization Piperacillin/  tazobactam suspected | - | - | 0 | 0 | 1 | Meropenem | - | - | Meropenem reexposed (tolerated) |
| 4 | trigger unknown | n/a | n/a | 2 | 2 | 1 | Fluconazole | n/a | n/a |  |
| 5 ^V^ | No proven sensitization Allopurinol suspected | - | - | 0 | 1 | 2 | 1. Amoxicillin /  Clavulanic acid  2. Vancomycin | -  -  - | -  -  - |  |
| 6 | Sulfamethoxazol | + | + | 0 | 0 | 2 | 1. Diclofenac 2. Daptomycin | + - | + n/a |  |
| 7 | Phenytoin Carbamazepin Amoxicillin | n/a + + | + + + | 0 | 0 | 3 | 1. Valproic acid 2. Clindamycin 3. Ceftriaxon | + - - | + n/a n/a |  |
| 8 | Sulfamethoxazol | + | + | 0 | 0 | 2 | 1. Vancomycin 2. Ceftriaxon | + - | + - |  |
| 9 | Ceftriaxon | + | + |  |  | 3 | 1. Iomeprol  2. Meropenem 3. Vancomycin | + - - | + - - | Meropenem and Vancomycin reexposed (tolerated) |
| 10 ^V^ | Meropenem Vancomycin | + - | - + | 1 | 1 | 2 | 1. Ciprofloxacin  Daptomycin 2. Pantoprazol  Fluconazol | - - - - | - - - n/a | Pantoprazole and Fluconazole reexposed (tolerated) |
| 11 | Vancomycin Rifampicin | - - | + + | 1 | 0 | 2 | 1. Iobitridol 2. Clindamycin | + - | + - |  |
| 12 | Carbamazepin | + | + | 0 | 0 | 1 | Flucloxacillin  (Cefuroxim) (Ceftriaxon) | +  +  + | -  -  - | All reexposed (not tolerated) |
| 13 | Piperacillin Meropenem Vancomycin | + - - | + + + | 0 | 0 | 1 | Sulfamethoxazole | - | n/a |  |
| 14 | Clarithromycin | + | - | 0 | 2 | 3 | 1. Gadobutrol 2. Omeprazole 3. Ceftriaxon | + -  - | n/a - - | Omeprazole reexposed (tolerated) |
| 15 | Phenytoin Lamotrigin Ceftriaxon Flucloxacillin | -  -  +  + | +  +  +  + | 1 | 0 | 1 | Iobitridole | + | + |  |
| 16 | Amoxicillin Ceftriaxon | +  + | n/a  n/a | 0 | 0 | 3 | 1. Vancomycin 2. Daptomycin  3. Norfloxacin | -  -  - | -  -  - | Vancomycin reexposed (not tolerated) |
| 17 | Dabrafenib | + | n/a | 0 | 0 | 1 | Zonisamid | n/a | n/a |  |
| 18 | Piperacillin | n/a | + | 0 | 0 | 3 | 1. Iomeprol 2. Levofloxacin 3. Metamizole | n/a - - | n/a n/a n/a |  |

Summary of all cases with drug-related relapses.

^V^ Viral reactivation, detected by PCR

LTT (Lympocyte transformation test), n/a (not applicable)
